# Gut microbiota composition in patients with Crohn’s disease in Saudi Arabia

**DOI:** 10.1371/journal.pone.0299749

**Published:** 2024-04-24

**Authors:** Hadil Alahdal, Ghaida Almuneef, Manal Muhammed Alkhulaifi, Omar Aldibasi, Abdulrahman Aljouie, Othman Alharbi, Zakiah Naser Almohawes, Fatemah Basingab, Mokhtar Rejili

**Affiliations:** 1 Department of Biology, Faculty of Science, Princess Nourah bint Abdulrahman University, Riyadh, Saudi Arabia; 2 Department of Botany and Microbiology, College of Science, King Saud University, Riyadh, Saudi Arabia; 3 Biostatistics Section, King Abdullah International Medical Research Center, Jeddah, Saudi Arabia; 4 King Saud bin Abdulaziz University for Health Sciences, Riyadh, Saudi Arabia; 5 Artificial Intelligence and Bioinformatics Department, King Abdullah International Medical Research Center, Jeddah, Saudi Arabia; 6 Department of Health Informatics, College of Public Health and Health Informatics, King Saud bin Abdulaziz University for Health Sciences, Riyadh, Saudi Arabia; 7 Department of Medicine, College of Medicine, King Khalid University Hospital, King Saud University, Riyadh, Saudi Arabia; 8 Department of Biological Sciences, Faculty of Science, King Abdulaziz University, Jeddah, Saudi Arabia; 9 Immunology Unit, King Fahad Medical Research Centre, King Abdulaziz University, Jeddah, Saudi Arabia; 10 Department of Life Sciences, College of Sciences, Al Imam Mohammad Ibn Saud Islamic University (IMSIU), Riyadh, Saudi Arabia; UAE University: United Arab Emirates University, UNITED ARAB EMIRATES

## Abstract

Crohn’s disease (CD) entails intricate interactions with gut microbiome diversity, richness, and composition. The relationship between CD and gut microbiome is not clearly understood and has not been previously characterized in Saudi Arabia. We performed statistical analysis about various factors influencing CD activity and microbiota dysbiosis, including diagnosis, treatment, and its impact on their quality of life as well as high-throughput metagenomic V3-V4 16S rRNA encoding gene hypervariable region of a total of eighty patients with CD, both in its active and inactive state with healthy controls. The results were correlated with the demographic and lifestyle information, which the participants provided via a questionnaire. α-diversity measures indicated lower bacterial diversity and richness in the active and inactive CD groups compared to the control group. Greater dysbiosis was observed in the active CD patients compared to the inactive form of the disease, showed by a reduction in microbial diversity. Specific pathogenic bacteria such as *Filifactor*, *Peptoniphilus*, and *Sellimonas* were identified as characteristic of CD groups. In contrast, anti-inflammatory bacteria like *Defluviitalea*, *Papillibacter*, and *Petroclostridium* were associated with the control group. Among the various factors influencing disease activity and microbiota dysbiosis, smoking emerged as the most significant, with reduced α-diversity and richness for the smokers in all groups, and proinflammatory *Fusobacteria* was more present (*p<0*.*05*). Opposite to the control group, microbial diversity and richness were lower in CD participants of older age compared to younger ones, and male CD participants showed less diversity compared to women participants from the same groups. Our results describe the first report on the relationship between microbiota and Crohn’s disease progress in Saudi Arabia, which may provide a theoretical basis for the application of therapeutic methods to regulate gut microbes in CD.

## Introduction

Crohn’s disease is a relapsing inflammatory illness mostly affecting the gastrointestinal tract [1]. It is characterized by abdominal pain, fever, and clinical indications of bowel obstruction or diarrhea with the passage of blood, mucus, or both [2]. The disease stage can be classified based on its activity and symptoms into two forms active and inactive; the active form has more severe inflammations. Despite CD being a chronic and incurable disease, remission is possible in some cases [3]. Since the pathogenesis of the disease, is suggested to be driven by complex interactions of genetic, environmental, immune, and microbial factors and manifests in a widely diverse clinical course, it is difficult to analyze disease mechanisms and predict disease development based on the patient’s condition at initial diagnosis [4].

Numerous cross-sectional studies support a pathogenic impact of the gut microbiota in CD. The gut microbiota, a complex community of bacteria, fungi, archaea, eukaryotes, and viruses residing in the mammalian gut, significantly influences host health [5]. Comprising one hundred trillion microorganisms and over a thousand bacterial species, the gut microbiota includes Firmicutes (*Bacilli* and *Clostridia*), Bacteroidetes, Actinobacteria (e.g., *Bifidobacterium*), and Proteobacteria (e.g., *Escherichia*) [6]. Concentrations vary along the gastrointestinal (GI) tract, with the stomach being the least populated due to acidity and the large intestine having the highest diversity [7]. Additionally, CD-associated dysbiosis was characterized by lower gut bacterial diversity as well as changes in the relative abundance of particular taxa, such as *Fusobacterium*, *Escherichia*, *Faecalibacterium*, *Roseburia*, *Ruminococcaceae*, *Peptostreptococcaceae*, *Christensenellaceae* and *Collinsella* [8].

Researchers face a formidable task when studying alterations in community composition in patients with CD due to the intricate diversity and abundance of the human microbiota, especially within the gut community. Using culture-independent techniques to explore bacterial diversity has significantly enhanced the understanding of gut microbiota composition. In the last decade, molecular techniques independent of cultivation, notably those using the small subunit ribosomal RNA (16S rDNA) gene, have provided a more comprehensive and less biased perspective on the gut microbiota [9, 10]. In the present study, we conducted sequencing of tags spanning the V4 and V3 regions to assess bacterial community composition and establish associations with disease activity. This approach allows for a more focused examination of specific taxonomic groups, facilitating the exploration of less abundant taxa, however, it shows a broader overview of community composition that might unveil critical microbial interactions [11–14].

This study aimed to compare the gut microbiota in CD and healthy Saudi participants and to correlate differences in the gut microbiota with various lifestyle variables. We performed 16S rDNA sequencing on stools from CD patients and healthy controls. The results were correlated with the demographic and lifestyle information, which were provided by the participants via a questionnaire. The findings of this study provide insights into the different stages of the disease and the intricate interplay between lifestyle variables and gut microbiota in healthy Saudi participants.

## Materials and methods

### Population characteristics

We performed a cohort study on the Saudi population to investigate microbial taxa associated with CD to understand the link between microbiota and CD progress and validate the outcomes with recent findings. We assessed the prevalence of Crohn’s disease in the City of Riyadh, the capital of the Kingdom of Saudi Arabia. It is in the center of the kingdom, with a population of 8.175.378 people at the time of data collection [15]. Samples were collected from patients treated at King Khalid University Hospital (KKUH). This public hospital receives patients from different regions of Saudi Arabia with an extensive IBD clinic and offers free health treatment to IBD patients. The protocols were submitted and approved by the National Committee of Bioethics (NCBE), Saudi Arabia. Ethical approval was obtained from the Institutional Review Boards Committee of Princess Nourah bint Abdulrahman University (reference, 21–0141). All volunteers received information concerning their participation in the study and gave written informed consent to sign their approval. Patients below 16 years of age were excluded as well as in case of treatment with antibiotics and/or probiotics during 1 month before collecting the stool sample, confirmed colostomy of the gastrointestinal tract, and lack of consent to participate in the study.

### Sample collection and subject characteristics

Eighty fecal samples (25 from patients with a previously confirmed active form of CD diagnosis, 40 from inactive cases of CD, and 15 from healthy people) are included in the setting of this work from June to August 2021. People treated at KKUH were identified according to the Diseases International Classification, 10^th^ revision (ICD-10) coding. Demographic data were recorded including different clinical variables (age, gender, body mass index, year of presentation, number of years of follow-up in the hospital, presenting symptoms, and major clinical findings on presentation). To study differences in the microbiome composition between CD and healthy patients, and between inactive and active disease (remission vs. recurrence), CD active and inactive patients were enrolled for a follow-up study. Inclusion criteria were a diagnosis of CD confirmed by endoscopy and histology in the past, and clinical remission—defined by the validated CD activity index (CDAI) for CD. Healthy participants (controls) had no previous history of chronic disease. At inclusion and during the follow-up (every 6–8 weeks), we collected diagnostic criteria, CD’s location, behavior, and clinical data including smoking and medical treatment. Clinical recurrence was defined by a value of higher than 150 for the Crohn’s Disease Activity Index (CDAI). Blood samples were collected to assess hematocrit HCT (blood cell count). Exclusion criteria included a severe concomitant disease involving the liver, heart, lungs, or kidneys, treatment with antibiotics during the previous 4 weeks, and pregnancy or breastfeeding for women. The most recently updated IBD phenotype details (according to the Montreal classification 11) were collected. Fecal samples were collected in sterile plastic containers (Sterilin U.K.) and brought to the laboratory in a freezer pack and stored at −80°C. Patients’ Characteristics and demographics are described in **Table 1**.

**Table 1:** Patients’ characteristics and demographics.

**Participants Groups**	**Total(n) %**	**Active(n) %**	**Inactive(n)%**	**Healthy people as control (n) %**
	80	25	40	15
	100%	31.25%	50%	18.75%
**Age** • < 16 (n) • 17–40 • Above 40	3 3.75% 57 71.25% 20 25%	1 4% 19 76% 5 20%	2 5% 31 77.5% 7 17.5%	- 7 46.7% 8 53.3%
**Gender (n)**	(49/31)	(13/12)	(28/12)	(8/7)
(Male/ Female)	61.25% / 38.75%	52% / 48%	70% / 30%	53.3% / 46.7%
**Body mass index (BMI) (mean)**	24.9145	22.86	26.252	24.774
**Crohn’s disease activity index CDAI (mean)**	-	219.76	74.8	-
**Smoking (n)**	(62 / 18)	(17 / 8)	(33/7)	(12/3)
(No/Yes)	(77.5% / 22.5%)	(71% / 29%)	(82% / 18%)	(80% / 20%)
**Hematocrit (mean)**	39.4 (2 groups)	37.092	40.8275	-
**Abdominal pain (n)** • No pain • Moderate • Sever	38 47.5% 26 32.5% 15 18.75% 1 1.25%	2 8% 9 36% 13 52% 1 4%	22 55% 16 40% 2 5%	14 93.3% 1 6.7% -
**Disease Location** (n) • L3 Ileocolonic • L2 Colonic • L1 Ilea • L4 Isolated upper disease+L2 Colonic • unknown	31 47.7% 12 18.5% 19 29.3% 2 3% 1 1.5%	11 44% 6 24% 7 28% 1 4% 0	20 50% 6 15% 12 30% 1 2.5% 1 2.5%	
**Disease behavior (n)** • B1 (non-stricturing and non-penetrating) • B2 (stricturing) • B3 (penetrating) • P (perianal involvement) • unknown	18 27.7% 10 15.4% 31 47.7% 16 24.6% 5 7.7%	7 28% 3 12% 12 48% 8 32% 2 8%	11 27.5% 7 17.5% 19 47.5% 8 20% 3 7.5%	

*n = Number of participants. *mean = the average number of every group.

### Disease activity and severity

CD patients were scored with the CDAI score during the follow-up visit. The CDAI consists of clinical parameters including general well-being, abdominal pain, number of liquid/soft stools per day, abdominal mass, hematocrit level, the use of anti-diarrheal drugs, standard weight, arthralgia; inflammation of the iris or uveitis; *Erythema nodosum*, *Pyoderma gangrenosum*, or aphthous ulcers; anal fissures, or abscesses; other fistulas, and fever (>100°F) during the previous week. Individuals who were in remission scored below 150 (CDAI <150) and those who were active scored above (CDAI ≥150).

### Amplification and sequencing of 16S rRNA encoding gene

Total DNA for gut microbiota analysis was extracted using the QIAamp Fast DNA Stool Mini Kit (Qiagen) following the manufacturer’s instructions. The DNA quantity and quality were measured for DNA concentration by spectrophotometer using Nanodrop QIAGEN model (QIAxpert) SN 200345. The V3-V4 hypervariable regions of the bacterial 16S rRNA gene were amplified with specific primers (Bakt_341F-805R) using Herculase II Fusion DNA Polymerase provided by Nextera XT Library Kit. The 16S rDNA metagenomic sequencing library preparation was performed based on the library Protocol. Samples were sequenced using an Illumina sequencer following the NGS library preparation procedure. For Metagenome, amplicon sequencing was performed with a Type of Read Paired-end 301 Read Length (Part # 15044223 Rev. B). Illumina SBS technology detects single bases as they are incorporated into DNA template strands using a proprietary reversible terminator-based method. Natural competition minimizes incorporation bias and reduces raw error rates, terminator-bound dNTPs are present during each sequencing cycle.

### Ligate adapters

The sequencing library was prepared by randomly fragmenting the DNA or cDNA sample. 5’ and 3’ adapter ligation follow. "Tagmentation", on the other hand, combines the fragmentation and ligation reactions into a single step, significantly increasing the efficiency of the library preparation process.

### Final library construction

Adapter-ligated fragments are finally PCR amplified using a PCR primer solution that anneals to each adapter’s end. The library templates are subjected to quality control and quantification processes.

### Cluster generation using bridge amplification

The library was loaded into a flow cell, and fragments were collected on a lawn of surface-bound oligos that complement the library adapters. Through bridge amplification, each fragment was then amplified into different clonal clusters. The templates were ready for sequencing after the cluster generation was complete.

### Data availability

Sequences were deposited in GenBank and accession numbers were obtained: PRJNA1053658.

### Statistical analysis

Statistical analyses were conducted in GraphPad Prism 9 and RStudio (R version 4.2.1). To normalize data, we analyzed the sequences from all groups with the upper quartile method of normalization (CITE) by following the Bioconductor workflow for microbiome data analysis [16]. To account for changes in library size and variance, scale, and microbiome count data need to be transformed. The transformation changes each sample’s counts into corresponding frequencies, also referred to as proportions or relative abundances. The amount of community difference from location to location within one habitat is approximated by β-diversity (as opposed to variation from sample to sample within one location). We analyzed the variance within each group to compare β-diversity across groups using “Adonis Test” permuted in PERMANOVA [17]. PERMANOVA (Permutational Multivariate Analysis of Variance) attempts to identify covariates that can explain the inter-subject variability observed by pairwise distances [18]. We used the mixed analysis of variance (ANOVA) to compare the three groups. A one-way ANOVA analysis of variance and two-way ANOVA for multiple comparisons were used for statistical comparisons to decipher associations between the demographic data (clinical metadata) such as age, body mass index (BMI), gender, smoking habits, abdominal pain, fistula, and disease location and severity. All values were presented as the mean, and standard deviation (SD). *P<0*.*05* was considered significant for all tests.

## Results

### Characteristics of the study population and disease phenotypes

We identified 65 prevalent cases of Crohn’s disease (Table 1); most of the patients were between the ages of 17–40 years (71.25%), and 24 patients were women (36.92%), and 41 were men (63.08%). Phenotypic details were available for the 65 CD cases (100%). Most patients with CD were diagnosed when aged 17–40 years and had the disease affecting both the ileum and colon (31 patients, 48%). The disease was inflammatory but non-structuring and non-penetrating (18 patients, 28%); but 31 patients, (48%) were classified as having a penetrating disease.

### Gut bacterial analysis

The overall microbiota of all groups was characterized by the presence of 4 phyla such as Firmicutes, Bacteroidetes, Proteobacteria, and Actinobacteria with the dominance of Fermicutes phylum. The major genera were *Phocaeicola*, *Bacteroides*, *Prevotella*, and *Escherichia/Shigella*.

### Taxonomic subset

Our results showed that Clostridiales was a taxonomic Order with a bimodal abundance profile in the data. Some pathogenic genera were presented in the two patient groups, but not in the control group, such as *Filifactor*, *Peptoniphilus*, and *Sellimonas* [19–21]. Other genera were found only in the control group and not in the two patient groups, such as *Defluviitalea*, *Papillibacter*, and *Petroclostridium*.

### Gut bacterial diversity and taxa common to CD patients and healthy people

**Fig 1:**
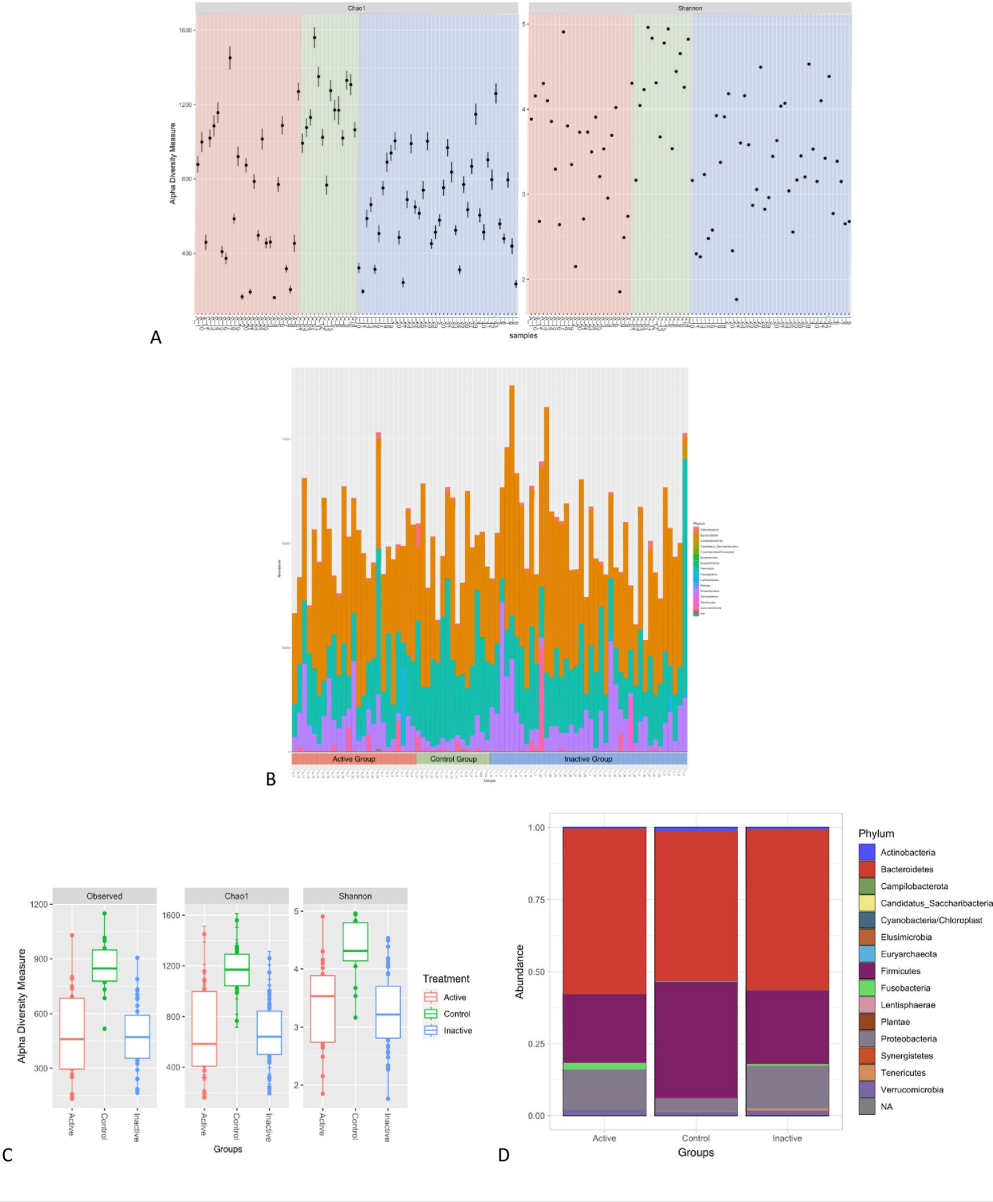
We visualize the different levels of taxa among different groups using a bar-plots. The dataset is plotted with each sample mapped individually to the horizontal (x) axis, and abundance values mapped to the vertical (y) axis. The abundance values for each OTU are stacked in alphabetical order. (A) α. diversity measure between samples of different groups (B) The most abundance phylum presents in every sample (C) boxplots of α-diversity richness estimators between different groups (D) The most abundance phylum presents in every group after we apply the mean for each group.

As shown by **Fig 1A,** our results reported that the Control group has more diversity and richness in both the Chao1 and Shannon diversity estimators. It was noted that in terms of α diversity, the inactive CD and control group had relatively similar alpha diversity (within the group) whereas the active CD group did not exhibit this homogeneity. Based on the visualization of the different levels of taxa among different groups using bar-plots, our results showed that the control group has a higher rate of Firmicutes compared to the rate of Proteobacteria and Bacteroidetes. There was a presence of Fusobacteria in the active group, and it appeared slightly in the inactive group, but it was not present at all in the control group (**Fig 1D**).

### β-diversity

**Fig 2:**
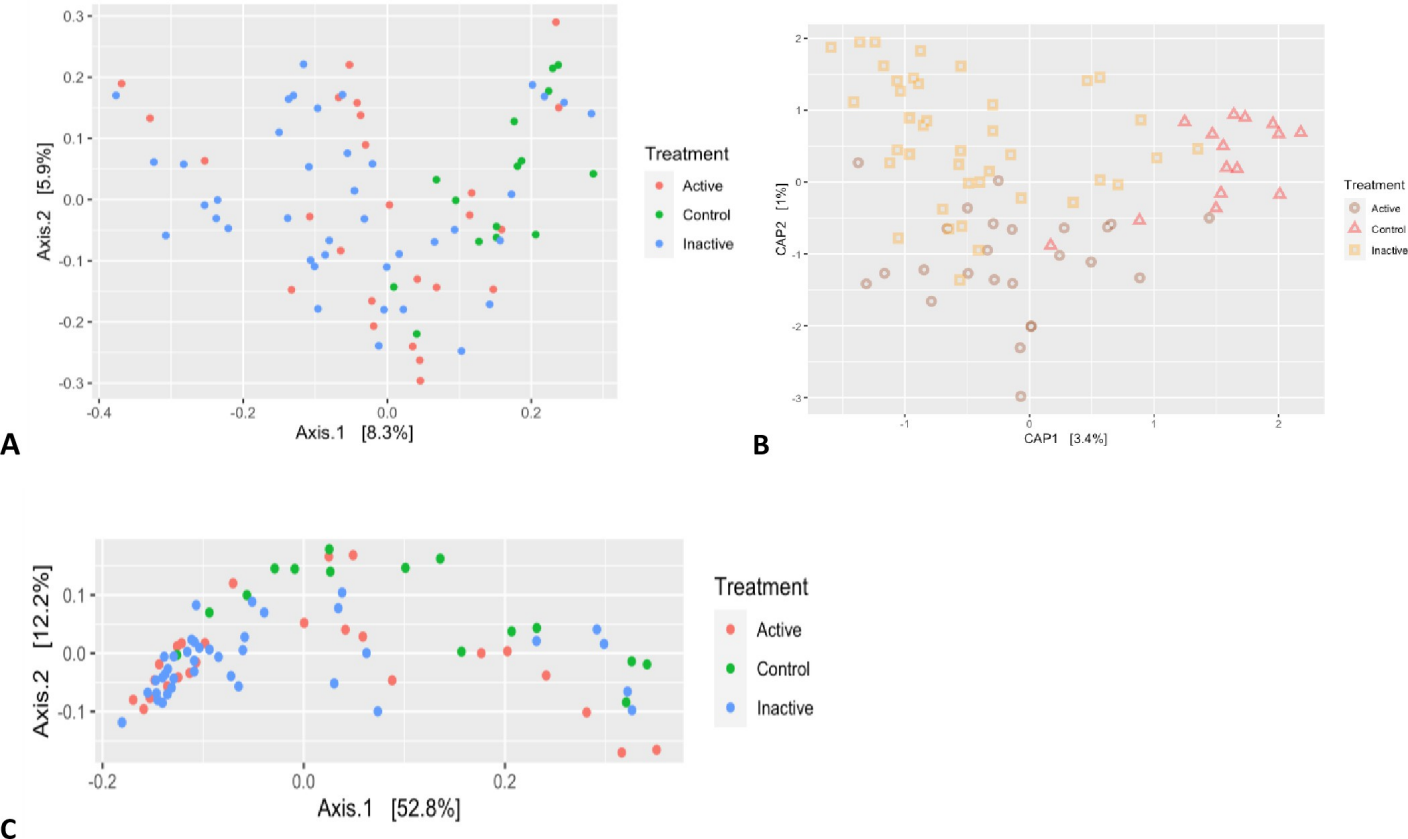
Between-sample dissimilarities were measured by (A) Bray-Curtis distance. (B) CAP-plot ordinate distances. The statistical significance was assessed using permutational multivariate analysis of variance (PERMANOVA) (Pr = 0.015; F = 3.9359; N.Perm = 999). (C) weighted UniFrac distances.

The bacterial community difference from location to location within one habitat is approximated by β-diversity. PERMANOVA analysis showed that the distribution and abundances of the three groups are significantly different (p < 0.015). A separation in community structure was reported between the Active CD, Inactive CD, and control group, suggesting a profound dysbiosis at disease severity (**Fig 2B**). Local contribution to β-diversity analysis confirmed that the microbiota structure of control group individuals differed from that of Active CD and Inactive CD with almost no difference shown between groups (**Fig 2A and 2C**).

### Factors influencing the gut microbiota composition for CD patients

#### Smoking

**Fig 3:**
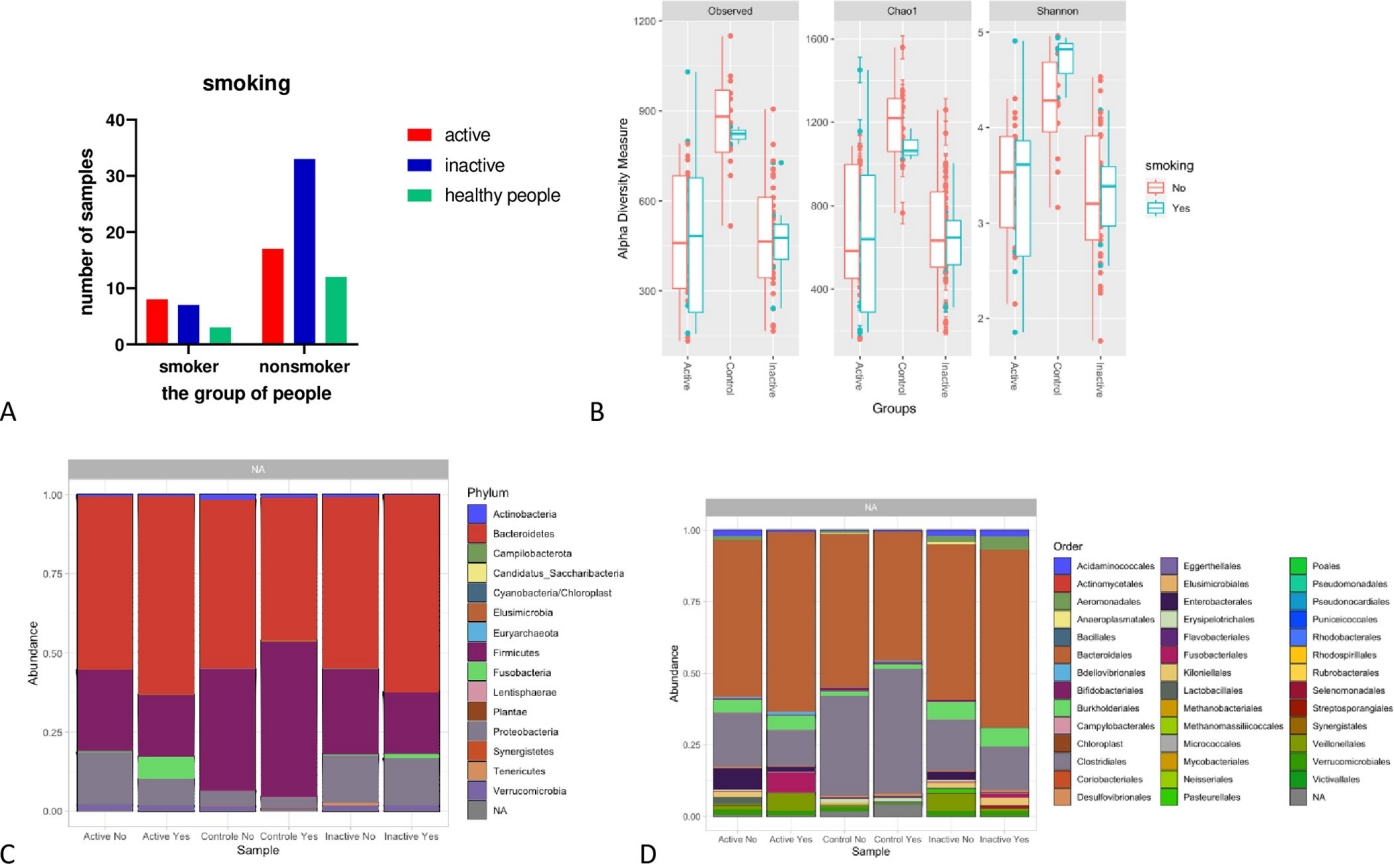
(A) Analysis of smoking and nonsmoking in Active CD, Inactive CD, and Control groups. (B) boxplots of α-diversity richness estimators between smokers and nonsmokers in the gut microbiota of patients with Active CD compared with Inactive CD and, healthy controls. (C) the most abundant phylum presents in every group, between smokers of each group. (D) the most abundant order present in every group, between smokers of each group. We visualize the different levels of taxa among different groups using a bar-plots. The dataset is plotted with each sample mapped individually to the horizontal (x) axis, and abundance values mapped to the vertical (y) axis. The abundance values for each OTU are stacked in alphabetical order.

The percentage of smokers in the active group was 29%, while it was 18% in the inactive group, and 20% in the control group represented in **Fig 3A** by the following numbers: n = 80 [Inactive Group n = 40 (smokers = 7, nonsmokers = 33), Active Group n = 25 (smokers = 8, nonsmokers = 17), and Healthy People n = 15 (smokers = 3, nonsmokers = 12)]. The nonsmokers had more richness and diversity in all the groups, despite the Shannon test showing higher diversity in the smoker’s control (**Fig 3B**). The phylum Fusobacteria was the highest in smoker patients, and the Firmicutes and Proteobacteria phyla were lower smoker patients than the nonsmoker’s ones (**Fig 3C**). Additionally, the order Enterobacterales was higher in nonsmokers compared to the nonsmoker’s group, and the order Fusobacteriales was higher in smokers’ group (**Fig 3D**).

#### Age

**Fig 4:**
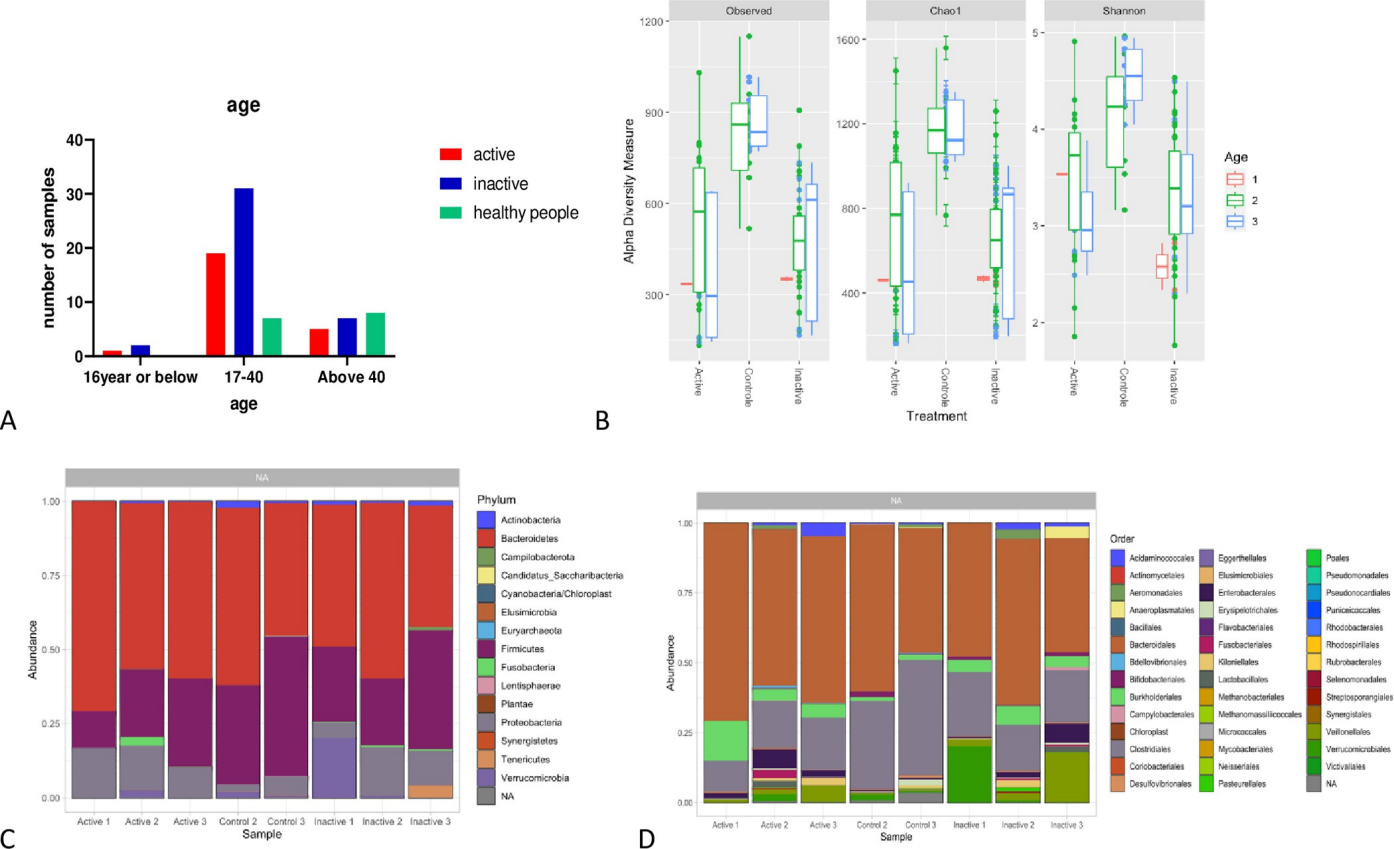
(A) The Age of participants in the Active and Inactive cases of CD and the Healthy people. (B) box plots of α-diversity richness estimators between different Ages (1 = < 16, 2 = 17–40 and 3 = Above 40) in the gut microbiota of patients with Active CD compared with Inactive CD and, healthy controls. (C) The most abundant phylum presents in every group after we apply the mean for each group, between the different Ages of every group sense (1 = < 16, 2 = 17–40 and 3 = Above 40). (D) the most abundant order present in every group after we apply the mean for each group. We visualize the different levels of taxa among different groups using a bar-plots. The dataset is plotted with each sample mapped individually to the horizontal (x) axis, and abundance values mapped to the vertical (y) axis. The abundance values for each OTU are stacked in alphabetical order.

The age factor has been found to have an impact on the structure of the gut microbiome. Alpha diversity plots demonstrated a slight increase in diversity as age increases in the control group. Most of the patients were between the age of 17–40 years (71.25%) as shown in **Fig 4A** by the following numbers: n = 80 [Inactive Group n = 40 (16 years or below = 2, 17–40 = 31, Above 40 = 7), Active Group n = 25 (16 years or below = 1, 17–40 = 19, Above 40 = 5), and control group n = 15 (17–40 = 7, Above 40 = 8)]. As reported in **Fig 4B**, participants who were above 40 had higher richness and diversity in the Inactive and control groups compared to the active groups. Interestingly, the phylum *Fusobacteria* was higher for patients with age (17–40). However, the *Firmicutes* phylum was higher in all groups at the age above 40 (**Fig 4C**).

#### Gender

**Fig 5:**
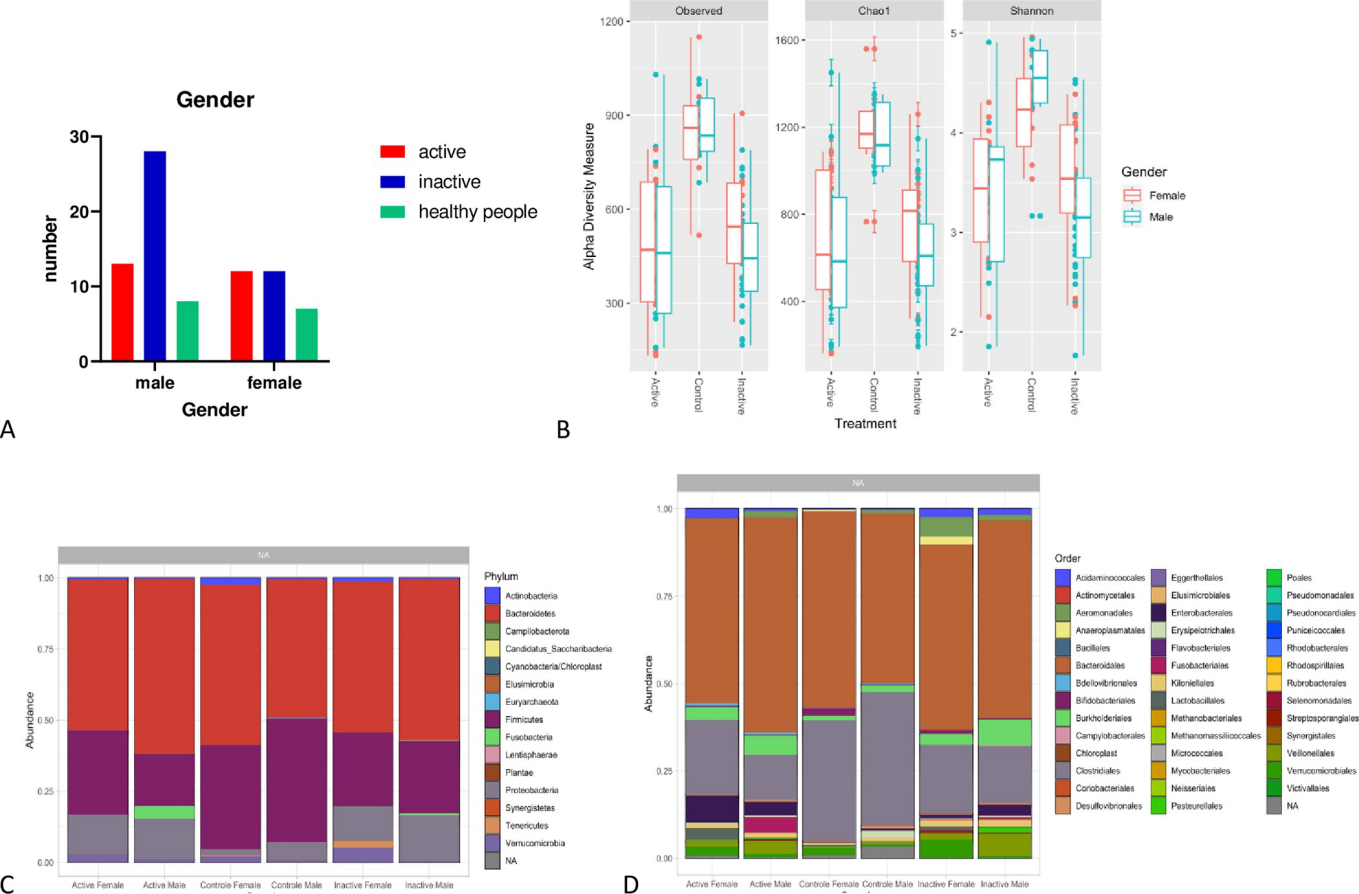
(A) Sex for participants with Active and Inactive Cases of Crohn’s Disease (CD) and Healthy People. (B) box plots of α-diversity richness estimators between different genders in the gut microbiota of each group. (C) bat plots show the most abundant phylum present in every group after we apply the mean from each group. (D) bat plots show the most abundant order present in every group. We visualize the different levels of taxa among different groups using a bar-plots. The dataset is plotted with each sample mapped individually to the horizontal (x) axis, and abundance values mapped to the vertical (y) axis. The abundance values for each OTU are stacked in alphabetical order.

The distribution of patients based on gender is presented in **Fig 5A**. Thirty-one participants were women (38.75%), and 49 were men (61.25%). Among the studied groups, the number n = 80 comprised of [(Inactive Group n = 40 (28 males and 12 females), Active Group n = 25 (13 males and 12 females), and control group n = 15 (8 males and 7 females)]. All the tests showed that males had more richness and diversity in the control group, however, in the females, the CD groups had more richness and diversity (**Fig 5B**). The male in the control group had higher Firmicutes, Proteobacteria, and Bacteroidetes phyla. Similarly, females with CD tend to have higher levels of Firmicutes and Proteobacteria with the presence of the Tenericutes phylum (**Fig 5C**).

## Discussion

In this study, we wanted to focus on characterizing the microbiota heterogeneity in CD patients in comparison to healthy controls for a better understanding of the clinically associated symptoms of the CD disease. Also, we investigated patients’ characteristics and environmental factors with disease severity and microbiota heterogeneity. As far as we know, this is the first report describing the association between CD and gut microbiome in Saudi Arabia.

From a microbial point of view, our results showed that the GI tract of CD patients in both remission (inactive group) and active state displayed dysbiosis. Many of the current research efforts are aimed at identifying specific microbial signatures [22]. In our study, proinflammatory Fusobacteria, and the species *F*. *nucleatum* were detected in both CD groups. The latter has been linked to human colorectal cancer in both patient populations and disease stages. Because it is frequently isolated and identified in anaerobic samples from patients with various infections, *F*. *nucleatum* has long been believed to be an opportunistic pathogen [23] Therefore, its presence at higher levels in the active group is an important indicator of the presence of inflammation.

In CD, the intestinal microbiota is characterized by decreased diversity, lower proportions of Firmicutes which have anti-inflammatory properties in both vitro and in vivo, and higher proportions of Proteobacteria and Actinobacteria. Furthermore, the microbiota of CD patients is overpopulated with proinflammatory bacteria (e.g., *Escherichia* and *Fusobacterium*) and underpopulated with anti-inflammatory bacteria (e.g., *Faecalibacterium*) [24–26].

In this study, the genus *Peptostreptococcus* including *Peptoniphilus* spp, was observed in the CD groups, which is known to be associated with bloodstream infection [27]. Additionally, the genus *Sellimonas* was observed in the CD groups. Although little is known about this member of the *Lachnospiraceae* family, it was found to be more abundant in patients who have regained intestinal homeostasis following dysbiosis occurrences [28]. Interestingly, the presence of *Sellimonas* in the inactive group was greater than in the active group, and this may be a kind of adaptation to restore intestinal homeostasis more effectively.

The genus *Filifactor* was also found in the two CD groups, with the species *F*. *alocis*, a formerly unidentified Gram-positive anaerobic rod. This bacterium is now recognized as a newly emerging pathogen that may play an important role in periodontal disease [29]. As for the *Cronobacter* genus, which was also in the two CD groups only. Several *Cronobacter* species have been associated with infections, and the intensity of virulence varies amongst strains. *Cronobacter* spp. associated with food pathogenies in all age groups including neonatal and infants [30, 31]. It was reported that severe *Cronobacter* infections encompass the attachment to host cell surfaces, followed by penetration of the intestinal and blood-brain barriers (BBB) [32]. Manifestations of this infection comprise symptoms such as fever and lethargy and can develop into conditions like conjunctivitis, bilious septicemia, urosepsis, or meningitis [33]

On the other hand, there were the *Defluviitalea* genus found only in the control group. Since the development of culture-independent methods, a growing number of ecological studies have revealed that the phylum *Firmicutes* is one of the most abundant and ubiquitous bacterial groups in anaerobic digesters. It is generally recognized that members of the phylum Firmicutes’ order Clostridiales (such as *Clostridium*, *Acetivibrio*, *Selenomonas*, and *Ruminococcus*) are among the most popular hydrolytic bacteria in anaerobic bioreactors, particularly in cellulolytic conditions [34]. Gut Firmicutes have been associated with fiber fermentation, and positive interaction with intestinal mucosa, promoting homeostasis [33].

In addition, when compared to the control group, there was a significant increase in the amount of Proteobacteria in the CD patients. Proteobacteria with their invasive and adhering traits may change the gut microbiota, interfere with host defenses, trigger a pro-inflammatory response, and ultimately lead to IBD [35]. Additionally, studies have linked the increase in Proteobacteria to many of the beneficial microbes getting displaced from the gut lining signifying a diseased state [36]. A recent study by Houri et al. identified Proteobacteria including *Klebsiella* and *E*. *coli* as a major source of functional variability in the gut, which was also found in our study [37, 38].

Dietary changes, emotional changes, physical activity, and environmental exposure can all influence the survival of specific groups of microorganisms, therefore altering the biotic composition of the gut [37]. It was found that individuals who undergo the same environmental conditions tend to have similar gut microbiota compositions [39]. Furthermore, it is known that microbiota differs depending on genetics, geography, age, and lifestyle factors such as nutrition, smoking, and physical activity. Thus, all research subjects must be exposed to the same environment to prevent biases when comparing CD patients to the control group [39–42]. In this study, our control group consisted of people who were considered healthy and did not suffer from any digestive problems, who were all Saudi nationals, had a similar health status, and were, in general, exposed to similar environmental factors and other common lifestyle factors that could affect the composition of the gut microbiota.

In addition to our main purpose to study microbiota heterogeneity in association with disease severity, we wanted to address the correlation between patients’ demographic factors, gut microbiota composition, and disease severity. We studied several factors to see their impact on the gut microbiota composition in association with disease level, including gender, age, and smoking. Smoking was the most well-studied environmental risk factor for CD, with negative effects on the mucosal barrier as well as increased intestinal permeability and disease susceptibility [30, 31] Smoking has been found to change the gut microbiota by elevating the pH value of the intestinal tract, enabling the growth of some bacteria, and leading to an imbalance of the intestinal flora composition [43, 44]. Among the smoking group, smokers were 47% of the CD groups. Our results showed a reduced α-diversity and richness for the smokers in all groups using the Observed and Chao1 richness estimator. Moreover, elevation in pro-inflammatory bacteria, such as bacteria belonging to the Fusobacteria phylum was observed. This was accompanied by a decrease in the abundance of bacteria belonging to the phylum *Firmicutes*, known to contain many anti-inflammatory bacterial species. The results were more clearly visible in the active group, as the number of bacteria belonging to the Fusobacteriales order was higher. It is worth noting that the CD groups also comprise the species *F*. *nucleatum*, which is considered an opportunistic pathogen [23]. Other studies found similar findings, as Benjamin et al. found increased levels of Bacteroides–Prevotella in smoking patients with CD compared with nonsmokers [45]. Another study indicated a reduced microbial gene richness and taxonomic diversity in smoking patients with CD [46]. Similar to our findings revealed a reduction in Faecalibacterium with known anti-inflammatory properties [26] in inflamed mucosal tissue from smokers with active CD compared with non-smokers [47]. Mechanistic pathways to explain the role of smoking in mucosal damage, changes in intestinal irrigation, impairment of the mucosal immune response, and the progression of the disease are needed [48].

Age and gender affect how the gut microbiota is distributed. Gender has been recently indicated as one of the most influence factors on gut microbiota composition [49–51]. Our results showed a difference when we addressed the gender and age factors. More than two third of the participants were between 17–40 years old, and we noticed that people older than 40 years had higher microbial diversity and richness than younger ones, which was supported by other research findings [52, 53]. However, this was the opposite in the CD groups, as lower microbial diversity and richness were observed in older age groups. This was in agreement with the finding of Xu. et al. [54], as individuals with advanced ages exhibited increased levels in proinflammatory genera and reduced levels of some beneficial genera. We also noticed differences in the microbial composition between different ages in the three groups. The Firmicutes/Bacteroidetes ratio often rises with age and is viewed as a marker of gut dysbiosis; a greater ratio indicates a more disordered microbiota composition [55]. This bacteria-to-human cell ratio is also differing between genders, with women generally having a larger ratio of bacteria than males [56]. In this study, women participants comprised 48% of active CD patients, while they were 30% of the inactive group. All measures showed a similar result of α-diversity, where CD groups were less diverse for males, despite control men had higher microbial richness. There was also a difference in the microbial composition between genders; Fusobacteriales was present only in men, which can be attributed to male participants being smokers.

Investigating the relationship between microbiome heterogeneity and the occurrence of Crohn’s disease is of great significance. Changes in the composition and diversity of the microbiome may contribute to the development of Crohn’s disease and chronic inflammation in the intestines [57]. By studying the microbiome in individuals with Crohn’s, researchers can identify specific microbial species or imbalances associated with the disease. This knowledge can be used to develop targeted therapies to restore a healthy bacterial balance and alleviate symptoms [58]. Using microbiome analysis, healthcare providers can identify specific microbial biomarkers or signatures associated with the disease, enabling them to intervene proactively.

## Conclusion

This study provides evidence that changes in Firmicutes, Proteobacteria, and Bacteroidetes phyla/species levels are associated with CD both Active and Inactive. Increasing in *Fusobacteria* phyla/species levels is a significant indicator for the severity of CD, as it was linked to the Active stage of the disease. Additionally, smoking was found to be a risk factor for the severity of CD. Men had less diversity and richness in CD groups compared to women and more proinflammatory bacteria. We found that the severity of the disease increased with age.

To our knowledge, this is the first study to use microbiome analyses from stool samples between active and inactive Crohn’s disease and healthy subjects based on using Crohn’s disease activity index CDAI. It is also the first study to use microbiome analyses of CD patients in Saudi Arabia. Our findings contribute to a better understanding of CD microbiome perturbations and the identification of potential diagnostic and therapeutic targets. Future research should focus on investigating the impacts of different microbial metabolites to understand the specific relationship between gut microbiota alterations and CD pathogenesis.

## Supporting information

S1 Data (XLSX)
